# Evaluation of cognitive and mobility function in geriatric dogs following treatment with stem cell and stem cell extracellular vesicles derived from embryonic stem cells: a pilot study

**DOI:** 10.3389/fvets.2025.1549870

**Published:** 2025-03-26

**Authors:** Tae-Yoon Kim, Nam-Hee Kim, Jin-A Chae, Hyun-Keun Oh, Seonghyun Yang, Jae-Bong Moon, Seon Mi Wi, Ju-Hyun An, Ji Min Yu

**Affiliations:** ^1^Bio Research and Development Center, Daewoong, Co., Ltd., Yongin, Republic of Korea; ^2^Department of Veterinary Emergency and Critical Care Medicine, College of Veterinary Medicine, Kangwon National University, Chuncheon-si, Republic of Korea; ^3^Daewoong Pet, Corp., Seoul, Republic of Korea

**Keywords:** canine, CCDR, cognitive dysfunction syndrome, degenerative musculoskeletal diseases, LOAD

## Abstract

**Introduction:**

Declining physical or mental health in older dogs can lead to changes in the dog's cognitive and musculoskeletal function. Regrettably, these degenerative changes cannot be remedied. In the present study, geriatric small dogs exhibiting cognitive and behavioral changes were treated with human embryonic stem cell-derived mesenchymal stemcells (ES-MSCs, *n* = 21) and mesenchymal stem cell-derived extracellular vesicles (ES-MSC-EVs, *n* = 21).

**Methods:**

Before and 2 weeks after treatment, the cognitive and mobility status of the dogs were assessed using theCanine Cognitive Dysfunction Rating (CCDR) and the Liverpool Osteoarthritis in Dogs (LOAD) scale. Additionally, safety assessments were conducted through blood tests such as complete blood count and serum chemistry.

**Results:**

Following an assessment of clinical symptoms and blood tests in both the groups receiving ES-MSC and ES-MSC-EVs treatments, no notable side effects were detected. Moreover, the questionnaire survey revealed that both groups showed alleviation in CCDR and LOAD scores following administration.

**Discussion:**

These findings suggest that ES-MSC and ES-MSC-EV treatments have the potential to be used as a therapeutic option for improving clinical symptoms of degenerative diseases such as canine cognitive dysfunction and degenerativemusculoskeletal diseases in elderly dogs.

## 1 Introduction

Aging is a complex process that results in the deterioration of most organs and tissues. Innovations in technology, healthcare, and nutrition have notably extended the average lifespan of both humans and animals. Recent demographic research on animals indicates a growing population of elderly dogs, along with a rise in age-related degenerative conditions such as canine cognitive dysfunction syndrome (CDS) and degenerative musculoskeletal diseases (DMDs) (1). Cognitive functions encompass mental processes such as perception, awareness, learning, and memory, enabling individuals to gather information about their surroundings and determine their actions. CDS is a neurobehavioral disorder that impacts older dogs and cats, marked by an age-related decline in cognitive abilities significant enough to impair functioning (2). DMDs encompass a range of age-related disorders affecting the musculoskeletal system, including osteoarthritis (OA), sarcopenia, and degenerative joint diseases, which are among the most prevalent. Dogs afflicted with these conditions often suffer from heightened pain, reduced range of motion, and functional impairments (3, 4). Both CDS and DMDs significantly impact the quality of life in senior dogs, yet there are no definitive treatments available. Only alternative care is currently administered to alleviate symptoms (5, 6).

In human medicine, various studies were conducted on stem cells and stem cell-derived extracellular vesicles therapies for age-related diseases. They contain anti-inflammatory and antioxidant components, promoting neurogenesis, angiogenesis, reversing fibrosis and blood-brain barrier repair, making them valuable in treating geriatric diseases (7–11). Especially, stem cell derived-EV have emerged as a cell-free therapeutic option, demonstrating efficacy in reducing adverse effects and serving as a promising tool in regenerative medicine for geriatric disease (12). Despite these studies, there is still a lack of diverse research on the clinical efficacy and safety of stem cell and stem cell derived-EV in veterinary medicine.

This study aims to assess the improvement in cognitive dysfunction and mobility impairment in geriatric dogs through the administration of human embryonic stem cell-derived mesenchymal stem cells (ES-MSCs) and mesenchymal stem cell-derived extracellular vesicles (ES-MSC-EVs) treatments. Additionally, the safety of ES-MSC and ES-MSC-EV in geriatric dogs will be evaluated using blood tests.

## 2 Materials and methods

### 2.1 The institutional animals care and use committee

This study was conducted following the protocols approved by the Institutional Animals Care and Use Committee of Daewoong Pharmaceutical, Republic of Korea, and in compliance with the authorized guidelines (Approval number: IACUC-24-047).

### 2.2 Inclusion and exclusion criteria

Forty-three dogs aged 11 years or older and weighing 3 to 12 kg whose owners were willing to participate in the study were initially recruited into the study. Before participating in the test, a thorough history taking, physical examination, neurology examination, and blood test were conducted. As a result, dogs taking medications related to cognitive impairment or having diseases that could be confused with cognitive impairment syndrome were excluded. Ultimately, 42 dogs participated in this test, and were randomly divided into ES-MSC administration group and ES-MSC-EV administration group.

### 2.3 ES-MSC culture and characterization

Embryonic stem cell-derived mesenchymal stem cells (ES-MSCs) were derived from human embryonic stem cells (ESC) at Daewoong Pharmaceutical Co., Ltd., following previously established protocols (13). Briefly, ESCs were cultured in embryoid body (EB) formation media for 14 days, resulting in the formation of cell aggregates measuring 150–300 μm in diameter. These aggregates were subsequently transferred to CELLstart-coated culture dishes (Thermo Fisher Scientific, USA) and differentiated in mesenchymal stem cell media for an additional 16 days to generate ES-MSCs. The ES-MSCs were seeded at a density of 3,500 cells/cm^2^ in T-flasks or HYPER flasks (Corning, USA) using StemPro^®^ MSC SFM medium (Thermo Fisher Scientific, USA) and maintained at 37°C with 5% CO_2_. Upon reaching ~70% confluency, the cells were enzymatically passaged using CTS-TrypLE™ (Thermo Fisher Scientific, USA) for four consecutive passages. The ES-MSCs utilized in this study were at passage 12.

Characterization of ES-MSCs was performed according to modified minimal criteria for MSCs, as defined by the International Society for Cellular Therapy (ISCT, 2006) (14). Flow cytometric analysis was conducted using a FACSVerse™ flow cytometer (BD Biosciences, San Jose, CA, USA) to confirm the expression of MSC-specific surface markers, including CD29, CD44, CD73, and CD105. Additionally, the differentiation potential of ES-MSCs was evaluated through tri-lineage differentiation assays into osteocytes, chondrocytes, and adipocytes using commercially available differentiation media (Thermo Fisher Scientific, USA), following the manufacturer's protocols.

### 2.4 Isolation of ES-MSC-EVs and characterization

We performed the isolation and characterization of ES-MSC-EVs considering the International Society for Extracellular Vesicles (ISEV) guidelines (15). In order to manufacture EVs from ES-MSC, ES-MSCs were thawed and cultured in using DMEM/F12 (Thermo Fisher Scientific, USA) supplemented 10% FBS (Thermo Fisher Scientific, USA) until the cells reached about 80% confluency. In the 3D culture system, the cultured medium was discarded and replaced by fresh CD293 medium (Thermo Fisher Scientific, USA), and collected every 24 h and changed into fresh medium for three sequential days. The harvested medium was pooled to isolate ES-MSC-EVs. ES-MSC-EVs were isolated by tangential flow filtration (TFF) system and were concentrated to ~10 folds.

For the Nanoparticle Tracking Analysis (NTA) analysis, extracellular vesicles (EVs) purified using TFF were used. The analysis was conducted using the PMX-130 Mono ZetaView instrument from Particle Metrix, and the built-in software, Zetaview ver 8.06.01, was utilized. The dilution factor for the EVs sample was determined through a pre-test (200x), and the sample was prepared at a volume of 1 mL for measurement. The software instrument parameters were set as follows: filter wavelength in scatter mode, sensitivity at 80.0, shutter speed at 100, and frame rate at 30 fps. Measurements were taken at 11 positions per run, with 2 cycles per position, and the concentration and size were averaged from three independent experiments. EVs isolated and concentrated using Tangential Flow Filtration (TFF) were observed using a TEM (FEI Tecnai 10, USA).

Flow cytometry was used to analyze the CD markers (CD9, CD63, CD81) of EVs. For EVs analysis, Magnetic Capture Beads (Fujifilm, 297-79701) and CD marker antibodies CD9 (BD Pharmingen, 555,372), CD63 (BD Pharmingen, 556,020), and CD81 (BD Pharmingen, 555,676) were used. The procedure for conjugating the antibodies, magnetic beads, and EVs followed the protocol provided by the manufacturer of the Magnetic Capture Beads (Fujifilm, 297-79701). The analysis was performed using a FACS Verse flow cytometer (BD, FACS Verse) and the BD FACSuite v1.0.6 software. The analysis was performed using a sample with DPBS instead of EVs as the control.

### 2.5 Study design for ES-MSC and ES-MSC-EV administration

**Figure 1:**
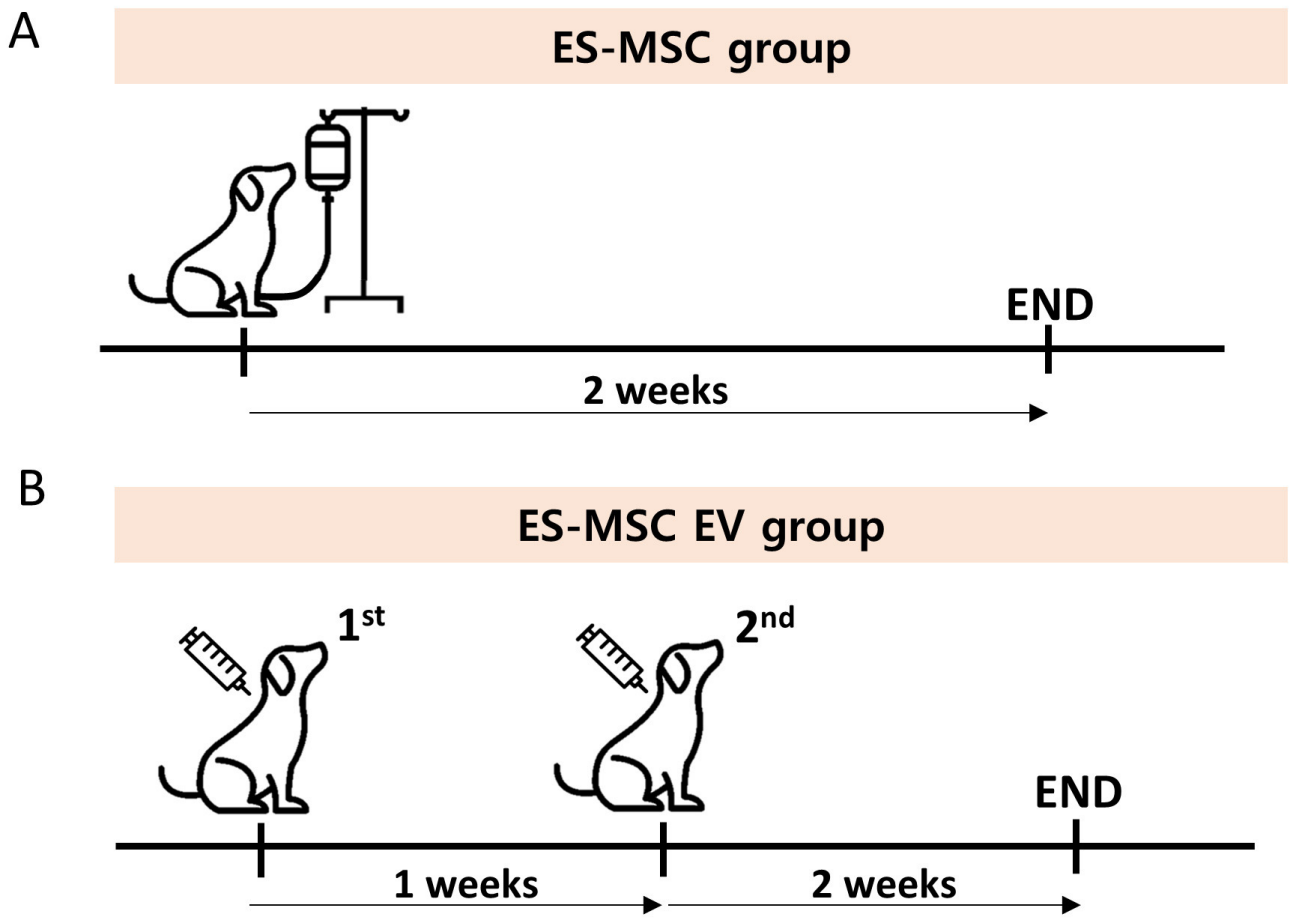
**(A,B)** Schematic diaphragm for this experiment.

This study was conducted at three animal hospitals: Helix Animal Hospital, Korean Animal Cancer Center, and Songjeong Animal Medical Center, all located in Seoul, Republic of Korea. Twenty-one dogs in ES-MSC group were injected intravenously with ES-MSCs only once. The dosage per injection was 1.0 × 10^7^ cells for dogs weighing 3–7 kg and 2.0 × 10^7^ cells for dogs weighing 7–12 kg. Then, these dogs were evaluated after 2 weeks (Figure 1A). Twenty-one dogs in ES-MSC-EV group received two administrations of ES-MSC-EV, on the first day and 1 week later. ES-MSC-EV was administered via subcutaneous injection. The dosage per injection was 1.0 × 10^10^ particles per dog, regardless of body weight. Then, these dogs were evaluated after 2 weeks (Figure 1B).

### 2.6 Evaluation safety of ES-MSC and ES-MSC-EV treatment in dogs

A comprehensive interview of the patient was conducted before and after treatment to assess appetite, activity, feces and vomiting. The scoring criteria used were as follow. appetite: 0 (normal), 1 (increased), −1 (decreased), −2 (severely decreased); activity 0 (normal), 1 (increased), −1 (decreased), −2 (severely decreased); feces 0 (normal), −1 (loose stool); −2 (diarrhea); vomiting 0 (normal), −1 (once a week), −2 (2–3 times a week), −3 (More than 3 times a week). To evaluate the safety of ES-MSC and ES-MSC-EV in both groups, the dogs were observed for any allergic reactions or anaphylaxis for 30 min after each injection. Blood tests, including complete blood count and serum chemistry, were performed before and after treatment to ensure the drugs did not impact kidney or liver function. Furthermore, to determine whether ES-MSC and ES-MSC-EV caused inflammation, serum levels of pro-inflammatory cytokines were measured using commercially available canine ELISA kits for interleukin (IL)-8 (Abcam, UK), IGF-1 (Neobiolab, USA) according to the manufacturer's instructions.

### 2.7 Assessment of cognitive dysfunction and mobility impairment

To assess the degree of cognitive impairment, the Canine Cognitive Dysfunction Rating (CCDR) (16, 17) questionnaire was conducted. The CCDR evaluates various issues related to memory (such as failing to recognize owners or house-soiling), orientation (like staring into space or getting lost in familiar surroundings), apathy (manifested by decreased activity or avoidance of interaction), impaired sense of smell (leading to difficulty in finding food), and locomotion. A summed CCDR score ≥50 is considered indicative of CCD. Just as with human dementia, it's crucial to rule out transient and reversible causes of behavioral changes before arriving at a definitive diagnosis. Therefore, the CCDR evaluation was performed by a veterinarian based on the owner's provided medical history. To evaluate the extent of mobility impairment, the Liverpool Osteoarthritis in Dogs (LOAD) (18) questionnaire was administered. The LOAD questionnaire is a 13-item clinical metrology instrument designed to evaluate canine articular disorders such as osteoarthritis. Scores from individual questions are combined to generate an overall “LOAD score,” which indicates the presence and severity of the animal's disease [phase 1 (Mild): 0–10; phase 2 (Moderate): 11–20; phase 3 (Severe): 21–30; phase 4 (Extreme): 31–52]. The LOAD questionnaire is completed directly by the owner.

### 2.8 Statistical analyses

Statistical analyses were conducted using GraphPad Prism version 6.01 software (GraphPad Software, CA, USA). Following the significant main effects found in the Two-Way analysis of variance (ANOVA), *post-hoc* comparisons were made using Bonferroni correction to account for multiple comparisons. Results are expressed as mean ± standard deviation. Statistical significance was defined as *P* < 0.05.

## 3 Results

### 3.1 Characterization of ES-MSC-EV

**Figure 2:**
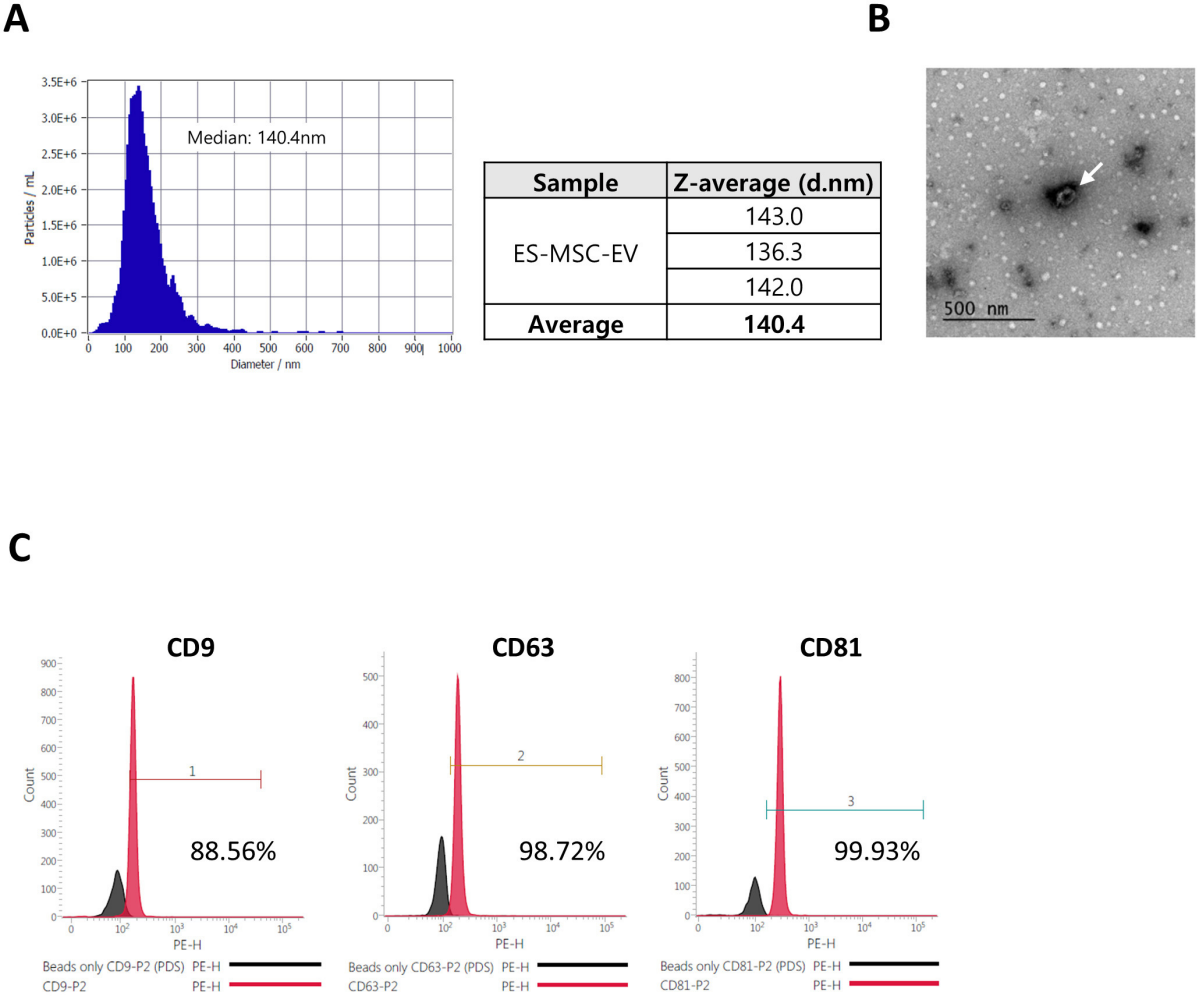
Characterization of mesenchymal stem cell derived extracellular vesicles (ES-MSC-EVs). **(A)** Particle size measurements by nanoparticle tracking analysis (NTA), **(B)** particle morphology and size measurements by transmission electron microscopy (TEM). Exosomes are indicated by arrows, and **(C)** analysis of cluster of differentiation (CD) marker (CD63, CD81, and CD9) by bead-capture flow cytometry.

ES-MSC-EVs isolated from ES-MSC and analyzed by nanoparticle tracking analysis (NTA), transmission electron microscopy (TEM) and bead-capture flow cytometry. The ES-MSC-EVs were within the normal range for EVs size (30–200 nm in diameter), and expressed CD9, CD63 and CD81 EVs markers, consistent with known characteristics of EVs and exhibited the characteristic spherical shape morphology (Figure 2). These results are consistent with the isolated EVs as being predominantly exosomes.

### 3.2 Characteristics of dogs participated in this study

The characteristics of 42 dogs that participated are summarized in Table 1. Maltese, Miniature poodles were most common breeds in both groups. Between ES-MSC and ES-MSC-EV group, there were no statistical differences in sex, age, body weight, CCDR score and LOAD stage. The most common concurrent diseases were myxomatous mitral valve disease (MMVD; *n* = 14), chronic kidney disease (*n* = 3), hyperadrenocorticism (*n* = 2), and hypertension (*n* = 2).

**Table 1:** Characteristic of patients in this study.

**Variables**	**ES-MSC group (*n* = 21)**	**ES-MSC-EV group (*n* = 21)**
Age, years^*^	13.9 ± 2.4 (11–19)	14.1 ± 2.5 (11–19)
Median age, years	14	13
Body weight (kg)^*^	4.8 ± 1.9 (2.4–11.0)	4.6 ± 2.2 (2.0–11.5)
Median body weight (kg)^*^	4.4	4.3
Sex (n)	CM (10), M (0), SF (8), F (3)	CM (6), M (0), SF (15), F (0)
Breed (n)	Miniature Poodle (5), Maltese (7), Shih-tzu (2), Bichon frise (1), Welsh corgi (1), Yorkshire terrier (1), Silky terrier (1), Mongrel (3)	Maltese (7), Miniature poodle (4), Pomerinain (2), Dachshund (2), Chihuahua (1), Chih-tzu (1), Miniature pinscher (1), Mongrel (2), Unknown (1)
Concurrent disease (n)	Myxomatous mitral valve disease (6), Hyperadrenocorticism (2), Chronic kidney disease (1), Porto-systemic shunt (1), Systemic hypertension (1), Protein losing enteropathy (1), Intervertebral disc disease (1), Ankylosis of spine (1), Lens subluxation (1)	Myxomatous mitral valve disease (8), Chronic kidney disease (2), Hypertension (2), Cognitive dysfunction (1), Immune-mediated thrombocytopenia (1), Tracheal collapse (1), Esophageal cancer (1), Stomach cancer (1)
CCDR score (n)	≥50 (*n* = 12), 40–49 (*n* = 3), < 40 (*n* = 4)	≥50 (*n* = 10), 40–49 (*n* = 10), < 40 (*n* = 1)
LOAD stage (n)	Stage 4 (*n* = 9), Stage 3 (*n* = 12)	Stage 4 (*n* = 15), Stage 3 (*n* = 4), Stage 2 (*n* = 2)

* Data are expressed as mean ± standard deviation. CCDR, The canine cognitive dysfunction rating scale; CM, Castrated male; EV, Extracellular vesicle; F, Female; LOAD, Liverpool Osteoarthritis *in* Dog; M, Male; MSC, Mesenchymal stem cell; SF, Spayed female.

### 3.3 Adverse effect and safety assessment following ES-MSC and ES-MSC-EV treatment

**Figure 3:**
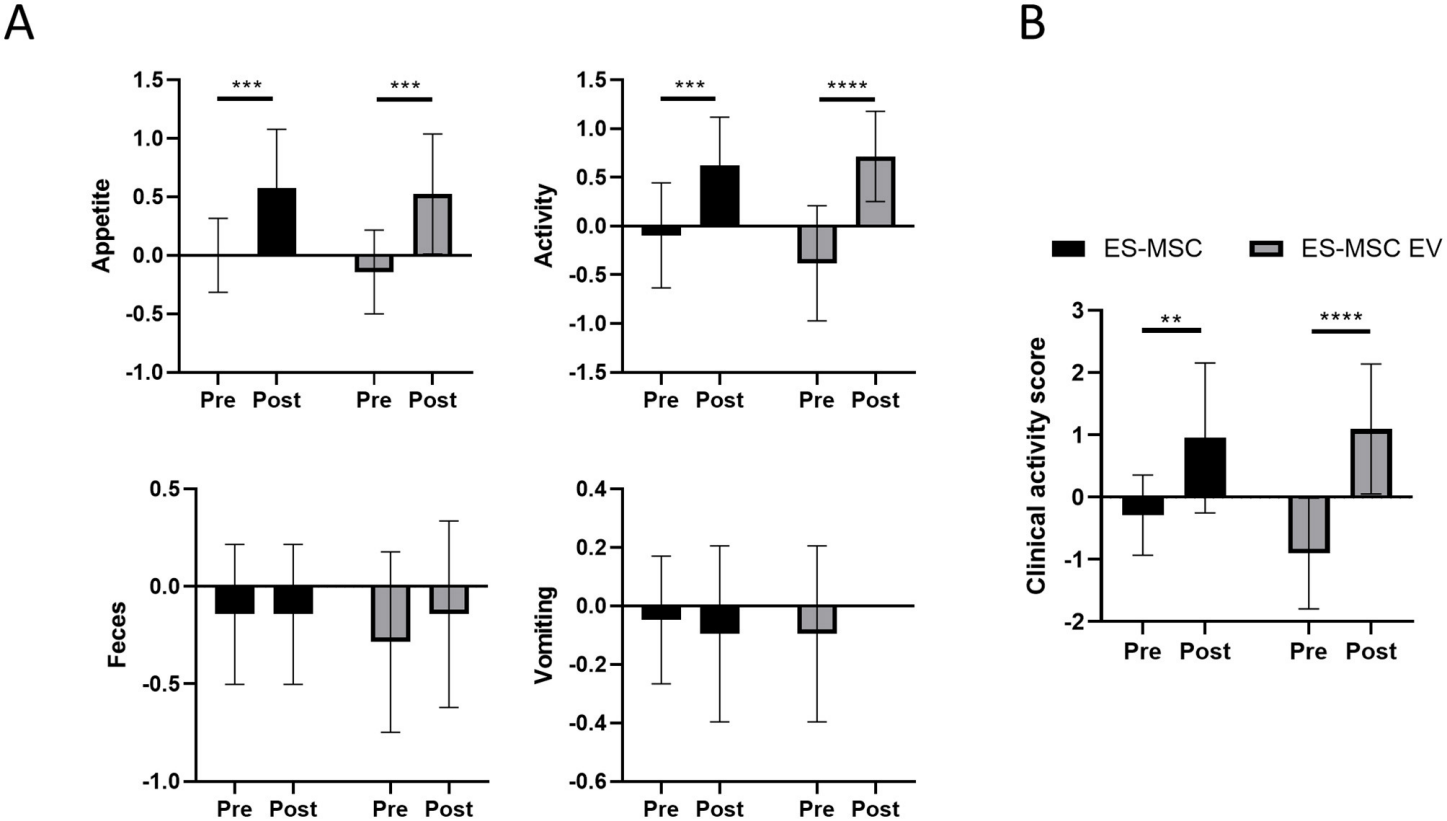
Evaluation of patient clinical symptoms including appetite, activity, feces, and vomiting after ES-MSC and ES-MSC-EV administration. **(A)** Appetite, activity, feces and vomiting scores before and after administration. **(B)** Clinical activity score, which is the sum of the scores of each activity indicator containing appetite, activity, feces and vomiting scores. ES-MSC, embryonic stem cell-derived mesenchymal stem cells; EV, extracellular vesicles. ^*^Value on the differences between before and after administration (^*^P < 0.05, ^**^P < 0.01, ^***^P < 0.001, ^****^P < 0.001). Results are represented as mean ± standard deviation.

As a result of monitoring for 30 min during ES-MSC and ES-MSC-EV administration, no abnormalities related to acute hypersensitivity reactions were identified in both groups. Two weeks after administration, the patient's clinical symptoms, including appetite, activity, stool, and vomiting, were checked. Significant improvements in appetite and activity were confirmed 2 weeks after administration (Figure 3A). As a result of adding up scores related to appetite, activity, stool, and vomiting and comparing them with before administration, a significant increase in clinical activity was confirmed in both groups. In particular, it was confirmed that there was a more significant improvement in the ES-MSC-EV administration group compared to the ES-MSC administration group (Figure 3B). Additionally, no significant differences were observed in hematological findings in both groups (Table 2). Additionally, to confirm whether administered ES-MSC and ES-MSC-EV cause inflammation in the body, pro-inflammatory cytokines IL-8 and IGF-1 were measured in serum. As a result, no significant difference was observed before and after administration in both groups (Table 3).

**Table 2:** Changes in complete blood count and serum biochemistry in geriatric dogs enrolled in the clinical trial using ES-MSCs and ES-MSC-EVs.

**Parameter**	**Reference range**	**ES-MSC group** **(*****n*** = **21)**		**ES-MSC-EV group** **(*****n*** = **21)**	
		**Before**	**After**	**Before**	**After**
RBC	5.7–8.8 (M/uL)	7.09 ± 0.67	7.10 ± 0.74	6.92 ± 1.25	6.93 ± 1.15
Hb	12.9–18.4 (g/dL)	16.21 ± 1.78	16.30 ± 1.75	15.95 ± 2.71	15.94 ± 2.22
HCT	37.1–57 (%)	52.73 ± 5.82	52.81 ± 6.07	50.57 ± 8.09	52.16 ± 7.31
MCV	58.8–71.2 (fL)	74.46 ± 4.81	74.44 ± 4.76	73.42 ± 5.61	75.73 ± 4.88
MCH	20.5–24.2 (pg)	22.87 ± 1.32	22.96 ± 1.22	23.14 ± 2.14	23.15 ± 1.63
MCHC	31–36.2 (g/dL)	30.76 ± 1.14	30.89 ± 1.11	31.51 ± 1.53	30.60 ± 1.52
RDW	11.9–14.5 (%)	13.05 ± 0.61	13.13 ± 0.89	13.13 ± 1.59	13.84 ± 3.88
WBC	5.2–13.9 (k/uL)	8.89 ± 2.57	9.07 ± 2.50	9.49 ± 4.58	9.00 ± 2.69
NEU	3.9–8.0 (k/uL)	5.90 ± 2.23	5.79 ± 2.20	6.38 ± 3.54	5.84 ± 1.92
LYM	1.3–4.1 (k/uL)	1.76 ± 0.64	1.92 ± 0.72	1.88 ± 0.60	1.88 ± 0.65
MONO	0.2–1.1 (k/uL)	0.64 ± 0.42	0.62 ± 0.36	0.66 ± 0.52	0.74 ± 0.63
EOS	0–0.6 (k/uL)	0.52 ± 0.45	0.65 ± 0.44	0.49 ± 0.38	0.48 ± 0.27
BASO	0–0.1 (k/uL)	0.02 ± 0.01	0.03 ± 0.03	0.03 ± 0.03	0.02 ± 0.01
PLT	143.3–400 (k/uL)	313.62 ± 166.96	320.71 ± 134.69	374.10 ± 176.38	379.95 ± 174.41
AST	10–51 (U/L)	44.50 ± 31.49	38.78 ± 20.66	32.30 ± 12.33	28.10 ± 7.24
ALT	17–111 (U/L)	139.29 ± 159.47	112.74 ± 74.96	71.06 ± 68.60	68.30 ± 53.35
ALP	17–111 (U/L)	340.19 ± 365.85	259.48 ± 298.93	149.67 ± 156.94	196.67 ± 376.15
GGT	17–98 (U/L)	13.18 ± 16.84	10.28 ± 8.02	8.14 ± 4.20	8.10 ± 3.37
BUN	21–124.8 (mg/dL)	23.00 ± 17.39	20.07 ± 12.23	28.48 ± 18.97	31.17 ± 25.68
CREA	0.3–1.5 (mg/dL)	0.89 ± 0.23	0.97 ± 0.28	1.15 ± 0.42	1.15 ± 0.44
TP	4.9–7.6 (g/dl)	6.65 ± 0.68	6.51 ± 0.46	6.17 ± 0.44	6.31 ± 0.44
ALB	2.3–4.2 (g/dl)	3.06 ± 0.39	2.99 ± 0.38	2.93 ± 0.29	2.98 ± 0.31
Globulin	1.9–4.5 (g/dl)	3.58 ± 0.65	3.66 ± 0.74	3.24 ± 0.35	3.33 ± 0.34
GLU	67–147 (mg/dL)	104.73 ± 10.55	109.49 ± 11.41	103.54 ± 19.16	183.40 ± 367.58
T CHOL	127–392 (mg/dL)	232.26 ± 56.66	229.25 ± 59.69	246.08 ± 64.99	256.94 ± 57.12
TG	21–124.8 (mg/dL)	149.13 ± 181.92	196.90 ± 211.83	122.38 ± 187.97	110.21 ± 145.04
T BIL	0–10 (U/L)	0.14 ± 0.06	0.15 ± 0.09	2.93 ± 0.29	0.12 ± 0.07
IP	2.4–6.4 (mg/dL)	3.87 ± 1.03	3.61 ± 1.00	3.95 ± 0.86	4.10 ± 0.99
Ca	8–11.7 (mg/dL)	9.93 ± 0.88	9.85 ± 0.92	9.83 ± 0.95	9.68 ± 0.65

Data are expressed as mean ± standard deviation. ALB, albumin; ALP, alkaline phosphatase; ALT, alanine aminotransferase; AST, aspartate aminotransferase; BASO, basophil; BUN, blood urea nitrogen; Ca, calcium; CK, creatinine kinase; Cl-, chloride iron; CREA, creatinine; EOS, eosinophil; GGT, gamma glutamyl transferase; Hb, hemoglobin; HCT, hematocrit; K+, potassium ion; LDH, lactate dehydrogenase; LYM, lymphocyte; MCH, mean corpuscular hemoglobin; MCHC, mean corpuscular hemoglobin concentration; MCV, mean corpuscular volume; MONO, monocyte; Na+, sodium ion; NEU, neutrophil; P, phosphorus; PLT, platelet; RBC, red blood cell; RDW, red cell distribution width; SDMA, symmetric dimethylarginine; T BIL, total bilirubin; TC, total cholesterol; TG, triacylglycerol; TP, total protein; WBC, white blood cell. ^*^Value on the differences between before and after treatment in each group (^*^P < 0.05).

**Table 3:** Level of inflammatory cytokines and insulin-like growth factor-1 in geriatric dogs enrolled in the clinical trial using ES-MSCs and ES-MSC-EVs.

**Parameter**	**ES-MSC group** **(*****n*** = **21)**		**ES-MSC-EV group** **(*****n*** = **21)**	
	**Before**	**After**	**Before**	**After**
IGF-1 (ng/ml)	52.01 ± 45.38	51.64 ± 60.93	52.76 ± 86.21	53.97 ± 85.85
IL-8 (pg/ml)	81.51 ± 48.91	81.60 ± 46.53	116.20 ± 187.47	83.55 ± 81.83

Data are expressed as mean ± standard deviation. IL-8, interleukin-8; TNF-α, tumor necrosis factor-α; IGF-1, insulin-like growth factor-1. ^*^Value on the differences between before and after treatment in each group (^*^P < 0.05).

### 3.4 Assessment of cognitive dysfunction after treatment with ES-MSC and ES-MSC-EV

**Figure 4:**
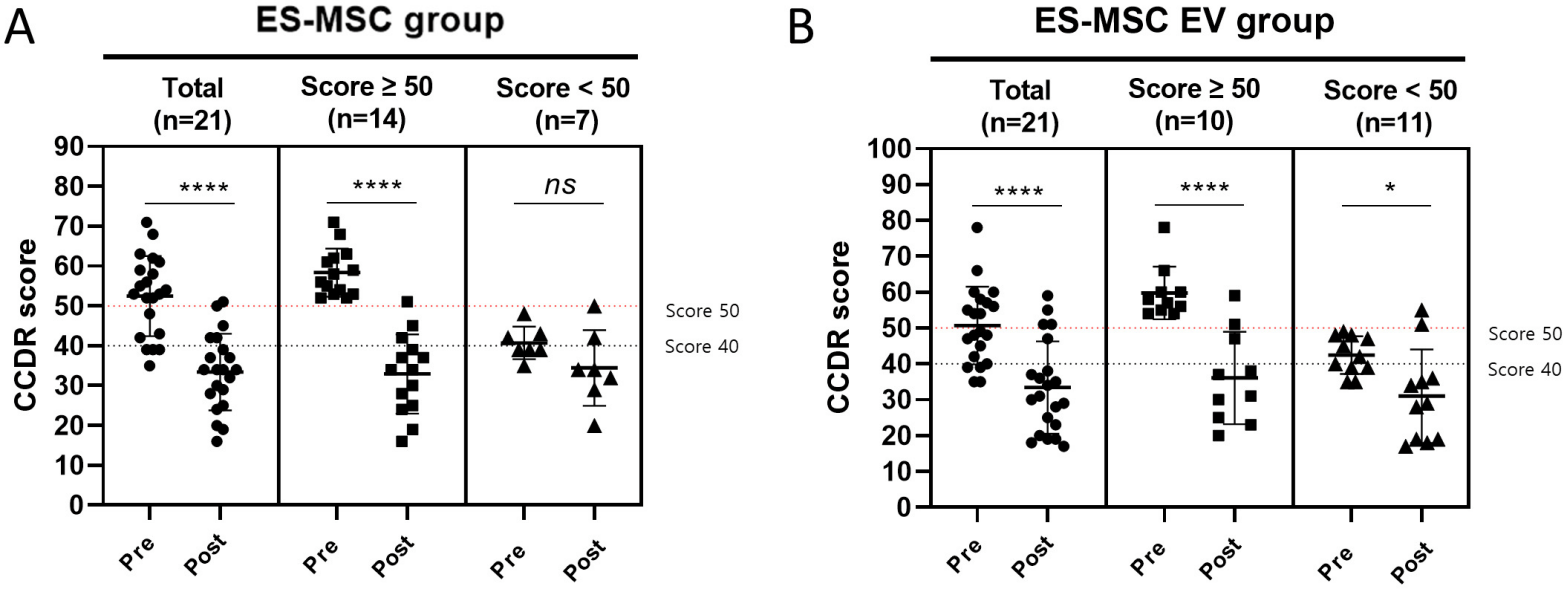
Changes in canine cognitive dysfunction rating (CCDR) score before and after administration. **(A)** Evaluation of CCDR score after administration of ES-MSC according to initial CCDR score. **(B)** Evaluation of CCDR score after administration of ES-MSC-EV according to initial CCDR score. ES-MSC, embryonic stem cell-derived mesenchymal stem cells; EV, extracellular vesicles. ^*^Value on the differences between before and after administration (^*^P < 0.05, ^**^P < 0.01, ^***^P < 0.001, ^****^P < 0.001). Results are represented as mean ± standard deviation.

CCDR scores were measured in each group before and after treatment (Figure 4). In the ES-MSC group, CCDR scores decreased in 19 dogs (19/21, 90.48%), with the mean score decreasing from 52.48 ± 10.06 before treatment to 33.43 ± 9.58 after treatment. In the ES-MSC-EV group, CCDR scores decreased in 18 dogs (18/21, 85.71%), with the mean score decreasing from 51.5 ± 10.45 before treatment to 34.25 ± 12.63 after treatment. Both groups were confirmed to have significantly improved scores after treatment. In particular, out of the total 42 dogs, 14 (14/21, 66.67%) were diagnosed with CCD in the ES-MSC group, and 10 (10/21, 47.62%) in the ES-MSC-EV group. All 14 dogs in the ES-MSC group showed a decrease in CCDR scores, with the mean score decreasing from 58.36 ± 6.01 before treatment to 32.93 ± 9.93 after treatment. Eleven dogs (11/14, 78.57%) improved to normal scores (≤39). Similarly, all 10 dogs in the ES-MSC-EV group showed a decrease in CCDR scores, with the mean score decreasing from 59.80 ± 7.35 before treatment to 36.1 ± 12.85 after treatment. Seven dogs (7/10, 70.00%) improved to normal scores (≤39). Notably, when ES-MSC-EV was administered, it was confirmed that the CCDR value was significantly improved not only in CCD patients with scores over 50, but also in patients with scores below 50 (Figure 4).

### 3.5 Assessment of mobility impairment after treatment with ES-MSC and ES-MSC-EV

**Figure 5:**
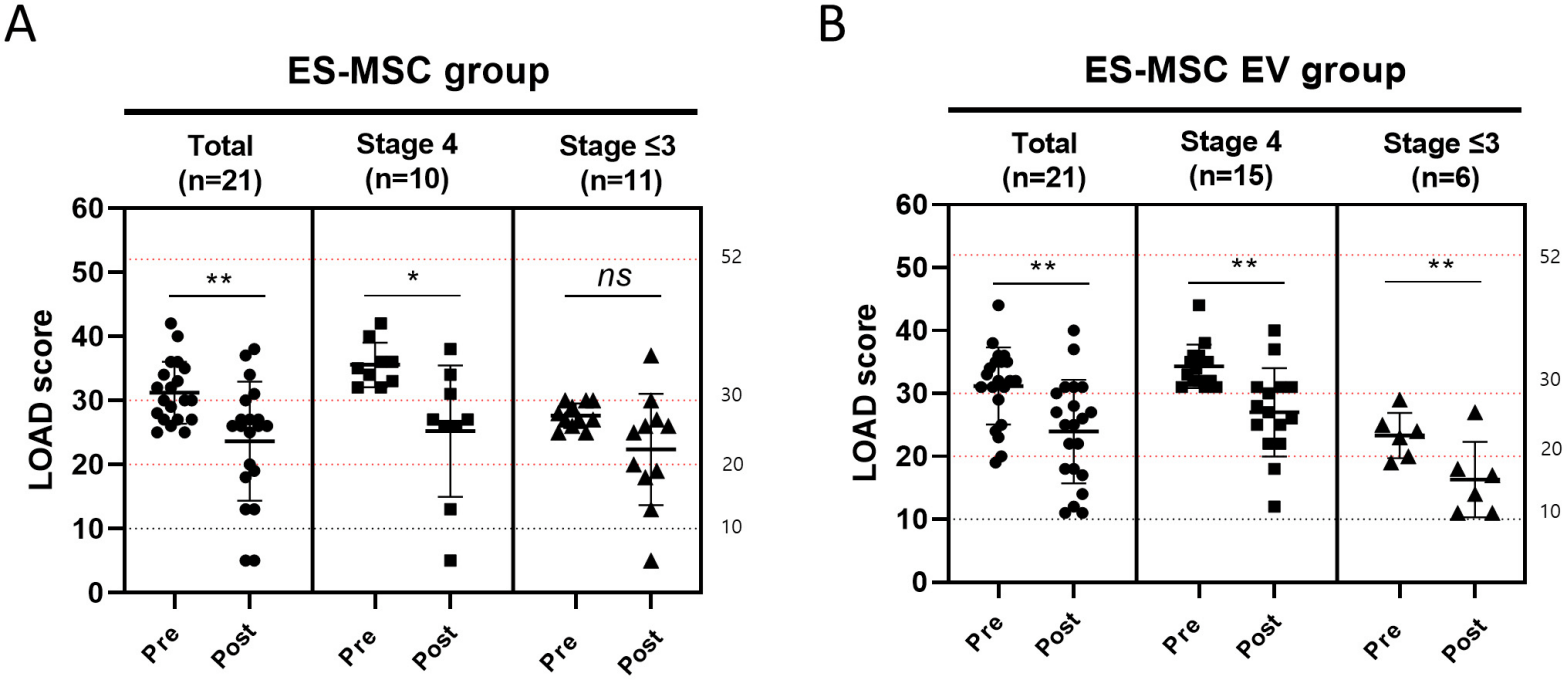
Changes in liverpool osteoarthritis in dogs (LOAD) score before and after administration. **(A)** Evaluation of LOAD score after administration of ES-MSC according to initial LOAD score. **(B)** Evaluation of LOAD score after administration of ES-MSC-EV according to initial CCDR score. ES-MSC, embryonic stem cell-derived mesenchymal stem cells; EV, extracellular vesicles. ^*^Value on the differences between before and after administration (^*^P < 0.05, ^**^P < 0.01, ^***^P < 0.001, ^****^P < 0.001). Results are represented as mean ± standard deviation.

LOAD scores were measured in each group before and after treatment (Figure 5). In the ES-MSC group, LOAD scores decreased in 17 dogs (17/21, 80.95%), with the mean score decreasing from 31.00 ± 4.80 before treatment to 23.14 ± 9.33 after treatment. In the ES-MSC-EV group, LOAD scores decreased in 18 dogs (18/21, 85.71%), with the mean score decreasing from 31.19 ± 6.12 before treatment (D0) to 23.95 ± 8.24 after treatment. For patients in Stage 4, it was confirmed that LOAD was significantly improved in both groups. However, for Stage 3 or lower, significant improvement in LOAD was confirmed only when ES-MSC-EV was administered (Figure 5).

## 4 Discussion

The findings of this study suggest that ES-MSC and ES-MSC-EV treatments may help alleviate clinical symptoms associated with cognitive decline and musculoskeletal degeneration in dogs. Administration of ES-MSC and ES-MSC-EV was associated with improved clinical ratings of cognitive and joint disorder behaviors. Furthermore, enhancements were observed in performance-based questionnaire evaluating cognitive and joint functioning in these dogs.

Previous studies have investigated the potential mechanisms underlying the efficacy of stem cells and stem cell derived extracellular vesicle (SC-EV) in age-related diseases at an experimental level. SCs and SC-EV are capable of producing various cytokines and neurotrophic factors, which support neuroregeneration. Consequently, a recent study reported that transplantation of SCs and SC-EV reduced Tau phosphorylation and inflammation in a mouse model of Alzheimer's disease (AD). Additional studies have demonstrated that SCs and SC-EV can decrease inflammation and enhance cognitive function in mice affected by AD. Furthermore, SCs and SC-EV contain members of the transforming growth factor superfamily (such as TGF-β1, TGF-β2, and TGF-β3) as well as several growth factors (including IGF-1, BMP-6, FGF-2, epidermal growth factor, and PDGF). These factors play a role in modifying the local pro-inflammatory microenvironment to promote tissue healing. Through these mechanisms, animal studies have illustrated that SCs and SC-EV can attenuate cartilage degradation, modulate subchondral bone remodeling, and foster cartilage regeneration. There have been several attempts to apply SC and SC-EV to aging-related diseases (regeneration, immune modulation, etc.) in human medicine (19, 20). In particular, a multicenter, open-label, single-arm, basket design clinical trial (NCT06607900) led by Zhang et al. (20) evaluated the safety and preliminary efficacy of SC-EV nasal drops in several neurodegenerative diseases, including Alzheimer's disease, Parkinson's disease, and frontotemporal dementia. Additionally, in a clinical trial study targeting osteoarthritis (NCT06688318) (20), the investigators aimed to investigate the effect of intra-articular injection of conditioned medium extracted from SCs in patients with knee joint OA aged 45 years or older, using functional scores and MRI with T2 mapping sequences. However, despite the extensive research conducted on these therapeutic mechanisms, which hold significant promise, treatments involving SCs and cSC-EV remain experimental to date. Therefore, this study aimed to assess the safety of administering ES-MSCs and ES-MSC-EVs to aged companion dogs. Additionally, by utilizing the CCDR and LOAD questionnaires, we evaluated not only the potential improvement in cognitive function in elderly dogs but also the alleviation of musculoskeletal disorders.

Referencing existing literature applied to canine subjects, ES-MSCs were administered intravascularly to dogs, while ES-MSC-EVs were injected subcutaneously into dogs, with each treatment evaluated separately. Furthermore, changes in blood analysis and clinical symptoms were monitored by assessing the CCDR and LOAD before and after the injections.

Previous research has indicated that dogs can develop CDS as they age, with the CCDR questionnaire commonly used to assess this condition (21). CCDR classify the severity of cognitive impairment based on behavioral signs such as disorientation, decreased interaction, sleep-wake cycle disturbances, house-soiling, decreased activity, and anxiety (22). In this study, both ES-MSC and ES-MSC-EV treatments resulted in a decrease in CCDR scores, confirming the improvement in CDS. Additionally, mobility in older dogs is a multifaceted issue influenced by various factors such as osteoarthritis (OA), age-related muscle atrophy (sarcopenia), and overall health (23, 24). LOAD is one of the main clinical metrology instruments used in veterinary practice to assess mobility impairment in dogs with chronic and degenerative musculoskeletal conditions (25, 26). In this study, both ES-MSC and ES-MSC-EV treatments were found to be effective in improving mobility impairment, as evidenced by a decrease in LOAD scores. Although the number of dogs with decreased LOAD scores was higher in the ES-MSC-EV group, the number of dogs with a reduction of more than one phase in LOAD scores was greater in the ES-MSC group. While these research findings did not directly compare ES-MSCs and ES-MSC-EVs, both treatments demonstrated the potential to decrease CCDR and LOAD scores. This suggests the possibility of utilizing them as novel treatments to alleviate age-related diseases in elderly dogs.

One limitation of our study is the lack of a placebo group, which might have resulted in subjective assessments by dog owners regarding their dogs' behavior. To address this, we conducted regular clinical check-ups, had the same veterinarian consistently evaluate the dogs' behavior, used a cognitive and musculoskeletal impairments questionnaire filled out by a family member or friend not living in the same household, and repeatedly performed problem-solving tests. Including a placebo group would have greatly strengthened the study's validity. However, finding older dogs without kidney or liver damage or other systemic diseases, and convincing owners to participate in testing a completely new substance, was challenging. Many owners were in distress, seeking help for their dogs, and numerous dogs were excluded due to common age-related medical conditions that could interfere with the treatment. With early detection of cognitive and musculoskeletal dysfunction by veterinarians and owners, and the preliminary safety data from our study, future research could involve more dogs and be placebo-controlled. In addition, our study confirmed the improvement of clinical symptoms of osteoarthritis after injection of ES-MSC and ES-MSC-EV, but did not confirm the improvement in radiographic imaging. Due to the small sample size, larger-scale studies are required in the future. In addition, the therapeutic effects of ES-MSC and ES-MSC-EV were not compared in this study, and further experiments are needed in this regard. We also did not determine which specific components of MSCs and EVs contributed to the therapeutic effects. In particular, SC-EVs are believed to play an important role in intercellular communication and have been investigated for their ability to deliver therapeutic cargo to target cells. In particular, in the case of extracellular vesicles, it is known that various components contained therein are delivered to recipient cells and exert effects. However, it should be noted that aged cells and EVs secreted from these cells can induce tissue inflammation because they can deliver factors associated with aging to recipient cells. Further studies are needed in this regard.

In conclusion, our results indicate that ES-MSC and ES-MSC-EV treatments improved clinical symptoms in dogs with cognitive decline and geriatric musculoskeletal issues without causing side effects. Overall, owners reported an enhancement in their dogs' quality of life. These findings suggest that ES-MSC and ES-MSC-EV treatments have the potential to be used as a therapeutic option for improving clinical symptoms of degenerative diseases in elderly dogs.

## Data Availability

The original contributions presented in the study are included in the article/supplementary material, further inquiries can be directed to the corresponding authors.
